# Patient-Reported Experience Across the Preoperative Ambulatory Trauma Pathway: A Prospective Cross-Sectional Study in a UK District General Hospital

**DOI:** 10.7759/cureus.112493

**Published:** 2026-07-11

**Authors:** Ghulam Dastagir Faisal Mohammed, Zaina Mansoor, Rachel Dhir, Siva Santhanam, Michael Clarke, Siwan Jones, Adit Ravishankar, Jebran Amin, Satya Pydah

**Affiliations:** 1 Trauma and Orthopedics, Ysbyty Gwynedd, Bangor, GBR; 2 Anesthesiology, Ysbyty Gwynedd, Bangor, GBR

**Keywords:** ambulatory trauma, day case surgery, orthopedic trauma pathway, patient-reported experience measures, patient satisfaction, preoperative fasting, surgical cancellation, waiting time

## Abstract

Introduction

Ambulatory trauma pathways manage a substantial proportion of operative orthopedic trauma, yet patient experience across these pathways is poorly characterized. This study aimed to describe patient-reported experience across two preoperative pathway domains, the ED attendance and the home-waiting interval, and to identify operational factors associated with poor experience.

Methods

This prospective survey included 65 consecutive adult ambulatory trauma patients at a UK district general hospital (October to December 2023). Patients rated their ED and home-waiting experience on a five-point Likert scale before planned surgery. Operational pathway metrics were captured prospectively via a digital handover platform and linked to experience scores. Low (≤3/5) and high (>3/5) experience groups were compared using the Mann-Whitney U test. Factors associated with low home-waiting experience were examined by logistic regression. All analyses were exploratory.

Results

Sixty-five patients were included (median age, 50 years; 60 (92%) self-presented; 50 (77%) were first assessed by an emergency nurse practitioner). Patients rating their ED experience as low (≤3/5; n = 22) had waited longer for first clinician review than those rating it as high (n = 43): median 91 vs. 31 minutes (p < 0.001) and had a longer total ED stay (5.4 vs. 4.0 hours, p = 0.027), whereas time to triage did not differ (p = 0.97). Patients reporting low home-waiting experience (27/65, 42%) had a longer ED-to-surgery interval (median 7 vs. 3 days, p = 0.018) and more operative cancellations (p < 0.001); 21 (32%) experienced at least one cancellation, with dissatisfaction rising from 27% among those never canceled to 75% among those canceled twice or more. On multivariable analysis, both a longer interval (adjusted OR, 3.31) and operative cancellation (adjusted OR, 4.91) were independently associated with low home-waiting experience. Forty-seven patients (72%) were uncertain about surgery timing, 13 (20%) received no red-flag advice, and only 17 (26%) had attempted to contact the trauma team during the home-waiting interval, with no dedicated contact route available; patients who had made contact reported a better home-waiting experience (p = 0.024).

Conclusions

Patient experience across the preoperative ambulatory trauma pathway was associated with time to first clinician review, total ED time, the ED-to-surgery interval, and operative cancellation, but not with triage speed or clinician grade. Uncertainty about surgery timing was the most prevalent problem. A structured preoperative communication package, including a dedicated point of contact, is a low-cost intervention that directly addresses the failures patients described.

## Introduction

Ambulatory (day-case) trauma accounts for a substantial proportion of operative orthopedic workload. The ORthopaedic Trauma Hospital Outcomes - Patient Operative Delays (ORTHOPOD) study, a multicenter prospective evaluation across 86 UK hospitals, found that day-case trauma patients accounted for 29.1% of the overall operative trauma burden, were predominantly working-age adults, experienced higher cancellation rates than inpatients (13.2% vs. 11.9%), and waited longer for surgery than inpatients with equivalent injuries [1,2]. Despite the widespread adoption of ambulatory pathways, little is known about patient experience between ED attendance and definitive surgery, a distinct interval during which patients wait at home, often with limited information and uncertain timelines.

Existing evidence demonstrates that waiting time influences patient satisfaction in orthopedic outpatient settings [3-5], but, to our knowledge, no published study has examined patient-reported experience across the full ambulatory trauma pathway, from ED attendance through the home-waiting interval to surgery. The British Orthopaedic Association has published timing standards for common ambulatory injury patterns [6], but compliance within a district general hospital (DGH) is constrained by competition for operating room capacity with hip fracture and other inpatient trauma workloads. The impact of these pressures on patient experience has not previously been evaluated.

Patient experience is now recognized as a central pillar of healthcare quality, alongside clinical effectiveness and patient safety, and is consistently associated with improved adherence, safety, and clinical outcomes across a wide range of settings [7]. Two features of the ambulatory trauma pathway make it a distinctive setting in which to study patient experience. First, operative cancellation on or close to the planned day is common. A global systematic review reported that elective procedures are canceled on the intended day of surgery in a substantial proportion of cases, most often for hospital- and capacity-related reasons rather than patient factors [8]. Such cancellations are not merely administrative inconveniences; patient-reported harm following canceled surgery includes physical deterioration, emotional strain, and loss of confidence in the service [9], and the burden is compounded when patients have already fasted. Second, prolonged or repeated preoperative fasting is itself a recognized source of harm and dissatisfaction, causing thirst, hunger, dehydration, and metabolic stress, and is frequently driven by fear of cancellation and unclear instructions rather than clinical necessity [10]. In the ambulatory trauma pathway, these two problems converge: a patient who is asked to fast and is then canceled experiences a wasted, burdensome attendance. Because this pathway predominantly affects working-age adults, in whom injury causes substantial and prolonged loss of productive time, fewer than two-thirds of injured UK adults have made even a partial return to work one month after injury, with upper-limb injuries associated with the greatest productivity loss [11], preoperative inefficiency carries an economic as well as a personal cost. Despite this, the patient-reported consequences of delay, cancellation, and poor communication in ambulatory trauma have not been characterized, and this gap is addressed by the present study.

The aims of this study were to describe patient-reported experience across the ED and home-waiting domains, to identify operational factors associated with poor experience, and to characterize communication failures during the home-waiting interval in order to propose targeted service interventions.

## Materials and methods

Study design and setting

This prospective cross-sectional survey was conducted as a service evaluation at Ysbyty Gwynedd, a DGH serving the population of North West Wales. Data were collected between October and December 2023. The ambulatory trauma operating list shares operating room capacity with hip fracture (neck of femur) and other inpatient trauma cases, which compete for the same daily sessions.

Participants

All adult patients (≥18 years) presenting to the ED during the study period with an injury subsequently requiring operative management without inpatient admission were eligible. Patients younger than 18 years and those requiring inpatient admission were excluded. Patients were identified consecutively from trauma meeting records and cross-checked against theater booking records.

Data collection

Operational pathway data were captured prospectively using an indigenous tool designed by the first author: THE LIST, a Microsoft SharePoint-based digital handover (Microsoft Corporation, Redmond, WA, USA) and patient management platform that is in routine clinical use across the trauma service. Recorded variables included time to triage, time to first clinician review, clinician type, total ED time, mode of presentation, the interval from ED presentation to surgery, and operative cancellation. Operative cancellation was defined as a planned procedure for which the patient had been instructed to fast and was then deferred, whether notified by telephone after fasting or sent home without surgery after attending while fasted, and therefore represents a fasted, nonproductive attendance rather than an administrative rescheduling of an unconfirmed date.

Survey instrument

Patients were surveyed before their planned surgery using a five-point Likert scale to rate their ED experience and home-waiting experience (1 = very poor, 5 = excellent), supplemented by binary items recording whether the reason for delay had been communicated, fasting instructions had been provided, whether the patient had attempted to contact the trauma team during the waiting period, whether operative cancellation had occurred (and how many times), and whether red-flag advice had been provided at ED discharge. Patients who could be reached by telephone were surveyed by phone; those who could not be reached were surveyed in person on admission, before surgery, so that experience data were obtained for the entire cohort. Open-text items captured the most significant concern during the waiting period. The instrument was developed locally and has not undergone formal psychometric validation.

Statistical analysis

Experience scores were dichotomized into low (≤3/5) and high (>3/5) groups. Continuous variables are reported as median (IQR) and compared using the Mann-Whitney U test with correction for ties; effect sizes are reported as r = Z/√N. Categorical variables were compared using the χ² test or Fisher’s exact test. Factors associated with low home-waiting experience were examined by univariable binary logistic regression. Predictors significant at p < 0.10 were entered into a parsimonious multivariable model, restricted to two predictors given 27 events, to maintain at least 10 events per variable. The ED-to-surgery interval was right-skewed and was dichotomized (>3 days) for regression. The significance threshold was p < 0.05; no correction for multiple comparisons was applied, and all findings are exploratory and hypothesis-generating.

Ethical considerations

The study was conducted as a service evaluation and did not require formal research ethics approval under the Health Research Authority decision tool. Verbal consent was obtained at the time of survey administration.

## Results

Cohort

Sixty-five adult patients met the inclusion criteria during the study period. The median age was 50 years (IQR, 33-60; range, 18-86), and 34 (52%) were female. Sixty (92%) self-presented to the ED, and 50 (77%) were first assessed by an emergency nurse practitioner (ENP). Triage category distribution and pathway characteristics are shown in Table 1.

**Table 1:** Cohort characteristics (n = 65). ENP, emergency nurse practitioner

Characteristic	n (%)
Age, years, median (IQR)	50 (33-60)
Female	34 (52)
Self-presented to ED	60 (92)
First assessed by ENP	50 (77)
Triage category 2	16 (25)
Triage category 3	22 (34)
Triage category 4	27 (42)

ED domain

Across the cohort, the median time to triage was 13 minutes (IQR, 8-22), the median time to first clinician review was 41 minutes (IQR, 22-98), and the median total ED time was 4.4 hours (IQR, 3.3-5.8). Patients rating their ED experience as low (≤3/5; n = 22) had waited substantially longer for first clinician review than those rating it as high (n = 43) and had spent longer in the department overall, whereas time to triage did not differ between the groups (Table 2). The dissociation between triage time and patient experience indicates that dissatisfaction tracked the wait for clinical contact and total ED stay rather than the speed of initial triage. Time to first clinician review and total ED time were themselves correlated (Spearman ρ = 0.53) and are best regarded as a single ED delay signal rather than independent factors. No association was found between ED experience and clinician grade (ENP vs. other; p = 0.35) or triage category.

**Table 2:** ED metrics by patient-reported ED experience (n = 65). * Mann-Whitney U test; U values are given in parentheses beside each p-value. Time to first clinician review was available for 64 patients. Statistical significance was set at p < 0.05.

Variable	ED experience ≤3/5 (n = 22)	ED experience >3/5 (n = 43)	p-value*
Time to triage, median (IQR), minutes	14 (9-20)	13 (8-22)	0.97 (U = 470)
Time to first clinician review, median (IQR), minutes	91 (47-159)	31 (14-58)	<0.001 (U = 712)
Total ED time, median (IQR), hours	5.4 (3.8-6.7)	4.0 (2.8-5.5)	0.027 (U = 633)

Home-waiting domain

Twenty-seven patients (42%) rated their home-waiting experience as ≤3/5, and 38 (58%) rated it as >3/5. The low-experience group had a longer interval from ED presentation to surgery (median 7 vs. 3 days, p = 0.018; Table 3). Twenty-one patients (32%) experienced at least one operative cancellation, and the distribution of cancellation counts differed significantly between experience groups (U = 722, Z = 2.78, p < 0.001; Table 4). Dissatisfaction increased with the number of cancellations, from 27% among patients who were never canceled to 69% after one cancellation and 75% after two or more cancellations.

**Table 3:** ED-to-surgery interval by patient-reported home-waiting experience (n = 65). * Mann-Whitney U test; the U value is given beside the p-value (U = 690, Z = 2.36). Statistical significance was set at p < 0.05.

Variable	Home-waiting ≤3/5 (n = 27)	Home-waiting >3/5 (n = 38)	p-value*
ED-to-surgery interval, median (IQR), days	7 (4-8)	3 (2-5)	0.018 (U = 690)

**Table 4:** Operative cancellations during the home-waiting interval, by patient-reported home-waiting experience. Values are n (%). Cancellation count was compared between groups using the Mann-Whitney U test, treating cancellation count as an ordinal variable (U = 722, Z = 2.78, p < 0.001). Statistical significance was set at p < 0.05. Percentages may not sum to 100 because of rounding.

Number of cancellations	Home-waiting ≤3/5 (n = 27)	Home-waiting >3/5 (n = 38)	Total (n = 65)
0	12 (44)	32 (84)	44 (68)
1	9 (33)	4 (11)	13 (20)
2	2 (7)	1 (3)	3 (5)
3	2 (7)	1 (3)	3 (5)
4	1 (4)	0 (0)	1 (2)
5	1 (4)	0 (0)	1 (2)

Communication and information provision

Communication failures were the most prevalent patient-reported problem across the pathway (Table 5). Forty-seven patients (72%) reported uncertainty about the timing of their planned surgery, and 13 (20%) reported receiving no red-flag advice at ED discharge. No dedicated route to contact the trauma team existed: patients who wished to make contact could do so only via the general hospital switchboard, often after delays or misdirected calls, and no staff member was designated to receive such calls. Only 17 patients (26%) had attempted to make contact during the home-waiting interval. Those who had made contact reported better home-waiting experience: three of 17 (18%) rated their experience as low, compared with 24 of 48 (50%) who had not made contact (Fisher’s exact p = 0.024).

**Table 5:** Communication and information provision (n = 65).

Measure	n (%)
Uncertain regarding timing of surgery	47 (72)
No red-flag advice provided at ED discharge	13 (20)
At least one operative cancellation while waiting	21 (32)
Contact number provided for home-waiting interval	17 (26)

Representative open-text responses illustrated the character of the home-waiting experience, with patients describing a lack of contact and uncertainty rather than the length of the wait itself: “Did not receive the expected call regarding the date of my surgery”; “Some days I was updated, some days there was no contact at all”; “I had to call every day to find out if there was space on the list”; and “No clear information about when surgery would happen - left in the dark.”

Factors associated with low home-waiting experience

On univariable logistic regression, an ED-to-surgery interval exceeding three days (OR, 4.81; 95% CI, 1.58-14.64; p = 0.006) and at least one operative cancellation (OR, 6.67; 95% CI, 2.10-21.18; p = 0.001) were each associated with low home-waiting experience. In a parsimonious multivariable model containing both variables, each retained an independent association: prolonged interval (adjusted OR, 3.31; 95% CI, 1.01-10.86; p = 0.048) and operative cancellation (adjusted OR, 4.91; 95% CI, 1.47-16.43; p = 0.010; model pseudo-R² = 0.18, likelihood-ratio p < 0.001). The model was limited to two predictors to preserve at least 10 events per variable (Table 6).

**Table 6:** Univariable and multivariable logistic regression for low home-waiting experience (≤3/5; 27 events, n = 65). Univariable p-values: interval, p = 0.006; cancellation, p = 0.001.

Predictor	Univariable OR (95% CI)	Multivariable adjusted OR (95% CI)	p-value (adjusted)
ED-to-surgery interval >3 days	4.81 (1.58-14.64)	3.31 (1.01-10.86)	0.048
Operative cancellation (≥1 vs. 0)	6.67 (2.10-21.18)	4.91 (1.47-16.43)	0.010

## Discussion

This study identified several factors associated with poor patient experience across the preoperative ambulatory trauma pathway: prolonged time to first clinician review and longer total time in the ED, an extended ED-to-surgery interval, and operative cancellation. Communication failure during the home-waiting interval, principally uncertainty about surgery timing, was the most prevalent patient-reported problem. We discuss these findings in order of service priority.

Communication failure during home-waiting

Nearly three-quarters of patients (72%) were uncertain about when their surgery would take place. Whereas the ED stage of the pathway has been the focus of system improvement initiatives, such as virtual fracture clinics, same-day emergency care, and dedicated ambulatory pathways, the home-waiting interval is typically left to ad hoc communication between the team and the patient. The open-text responses suggest that patients were distressed less by the length of their wait than by its unpredictability. Notably, no dedicated contact route existed: the only means of reaching the team was through the general hospital switchboard, with its attendant waits and misdirected calls, and no staff member was designated to respond. Yet the quarter of patients who nonetheless managed to make contact reported a significantly better home-waiting experience (18% vs. 50% dissatisfied, p = 0.024); whether a delay had been explained, by contrast, showed no such association, suggesting that patients wanted a date and a point of contact rather than an explanation. That even contact achieved through this unsupported route was associated with benefit makes the case for a proper, dedicated trauma team contact route more compelling, not less. This accords with evidence that better patient experience is linked to safer, more effective care [7] and that uncertainty and poor communication are themselves drivers of dissatisfaction independent of clinical outcome. Unlike operating room capacity, this is modifiable at negligible cost.

The 20% rate of absent red-flag advice warrants separate attention. In patients awaiting fixation of injuries with the potential for compartment syndrome, neurovascular compromise, or unrecognized progression, safety-net advice is a clinical governance requirement rather than an optional courtesy, given the established link between the quality of patient communication and patient safety [7].

Formalizing the trauma coordinator role

The communication failures identified point to a structural gap rather than individual shortcomings. In most units, coordination of the ambulatory waiting list falls to on-call junior doctors or part-time nursing staff, who are not continuously available and whose attention is necessarily divided. Maintaining real-time awareness of operating room availability, contacting patients about timing and fasting, and rescheduling canceled cases is a substantial, time-sensitive communication task ill-suited to ad hoc coverage. The value of a dedicated coordinator in trauma care is recognized: such roles center on timely communication with patients and families and on coordination among multidisciplinary teams and are regarded as a means of improving patient experience and continuity of care [12]. The patient navigator literature similarly associates a single, consistent point of contact with improved communication, care continuity, and patient satisfaction [13]. Our findings suggest that this work is consequential enough to warrant a dedicated, ideally full-time trauma coordinator responsible for the home-waiting interval, rather than being absorbed into existing on-call commitments.

Pathway time and cancellation

A longer ED-to-surgery interval and operative cancellation were each independently associated with low home-waiting experience, and dissatisfaction rose with each additional cancellation. The strength of the association with cancellation is best understood through what a cancellation entailed in this pathway: each represented a fasted, nonproductive attendance; the patient had fasted overnight and either traveled to the hospital or awaited a confirmatory call, only to be sent home without surgery. Repeated cancellations therefore impose repeated fasting, repeated disruption, and a cumulative erosion of confidence in the service, which plausibly explains the steep dose-response relationship observed. This is consistent with wider evidence that canceled surgery causes measurable patient-reported harm, such as physical deterioration, emotional strain, and loss of trust [9], and that prolonged or repeated preoperative fasting independently causes discomfort and metabolic stress, often precipitated by the very fear of cancellation seen here [10]. Because the ambulatory list competes for operating room capacity with hip fracture and other inpatient trauma, ambulatory cases are vulnerable to displacement when inpatient demand is high, and most day-of-surgery cancellations are driven by such hospital- and capacity-related factors rather than patient factors [8], which is the likely mechanism underlying both the delays and the cancellations patients described. These associations are cross-sectional and cannot establish causation; nonetheless, they suggest that pathway configurations delivering ambulatory trauma surgery promptly and with minimal cancellation (e.g., through ring-fenced or protected ambulatory operating room capacity or by confirming operating room availability before asking patients to fast) may be associated with a higher proportion of patients reporting a satisfactory experience.

These associations also argue for an explicit departmental agreement governing when patients are asked to fast or travel to the hospital. Patients should not be instructed to fast overnight or travel with an injured limb when there is material uncertainty about whether their operation will proceed. Although guaranteeing an operating slot in advance is not always practical, the pathway can be streamlined to reduce wasted attendances, for example, by confirming likely operating room availability before instructing a patient to fast and by adopting the principle that, once a patient has been asked to attend and has done so, the operating list should make every reasonable effort to accommodate them rather than displace them because of inpatient demand. Such arrangements shift the cost of capacity pressure away from the patient, who currently bears it through repeated fasting and futile journeys.

Time to first clinician review in the ED

Patients reporting low ED experience had waited longer for first clinician review (median 91 vs. 31 minutes, p < 0.001) and spent longer in the department overall (5.4 vs. 4.0 hours, p = 0.027), whereas neither triage speed nor clinician grade was associated with experience. Time to first clinician review and total ED time were correlated and represent a single ED-delay construct rather than two independent levers. Where operational pressure prompts consideration of changes in grade mix, these data suggest that investment in throughput, reducing the time to first clinical contact, is more relevant to patient experience than the grade of clinician providing that contact. The comparison by clinician grade rests on small numbers of non-ENP clinicians and should be interpreted as showing no association rather than evidence of equivalence.

Service recommendation: a structured preoperative communication package

The communication failures identified are coherent enough to be addressed by a single low-cost intervention: a written ambulatory trauma information package provided at ED discharge. Such a package should include an indicative surgery window with guidance on what to expect if rescheduling is required; a dedicated direct contact number for the trauma coordinator, with stated hours of availability; standardized preoperative fasting instructions, including what to do if surgery is delayed or canceled; a written list of red-flag symptoms warranting immediate return to the ED, in lay language; and practical home-waiting guidance covering analgesia, immobilization, weight-bearing status, and activity restrictions.

This intervention can be produced within existing patient information governance frameworks at minimal cost and directly addresses the failures patients described. The observed association between contact with the team and better experience provides preliminary support. Its effect should be evaluated prospectively.

Strengths and limitations

Strengths include the prospective design, the simultaneous capture of operational metrics and patient experience, complete experience data for the entire cohort (achieved by surveying in person those who could not be reached by telephone), and the timing of the survey before surgery, capturing the home-waiting domain at its natural endpoint.

Several limitations apply. This was a single-center study conducted over a three-month autumn-winter period, limiting generalizability across settings and seasons. The cohort is conditional on reaching surgery: patients who exited the pathway beforehand are not represented, and experience scores may therefore be biased toward more favorable ratings. Surveying patients shortly before surgery, sometimes by a member of the clinical team, may have introduced social desirability bias, and the ED domain was rated by recall, which may have been influenced by the waiting times against which it was compared. The instrument was locally developed and not psychometrically validated. Experience ratings were dichotomized at ≤3/5, which was a pragmatic rather than validated threshold, and the ED-to-surgery interval was dichotomized for regression because of skewness. Although based on complete data, the logistic model was limited by 27 events to two adjusted predictors and should be regarded as exploratory, with wide confidence intervals and possible residual confounding. Multiple comparisons were made without correction. Operative episode and postoperative experience were not captured.

## Conclusions

In this prospective single-center survey of 65 adults, patient experience across the preoperative ambulatory trauma pathway was associated with time to first clinician review, total ED time, the ED-to-surgery interval, and operative cancellation, but not with triage speed or clinician grade. A longer interval and operative cancellation were independently associated with home-waiting dissatisfaction, which increased with each additional cancellation. Uncertainty about surgery timing, reported by nearly three-quarters of patients, was the most prevalent and most readily modifiable failure, and patients who managed to make contact with the team, despite the absence of a dedicated route, reported a better experience. A structured preoperative communication package incorporating a dedicated point of contact, standardized fasting guidance, red-flag advice, and an indicative surgery window directly addresses these failures and should be evaluated prospectively.
